# Supplementary material for: Hyperchloremia and Hypernatremia Decreased Microglial and Neuronal Survival during Oxygen–Glucose Deprivation/Reperfusion
Source: Biomedicines. 2024 Feb 29;12(3):551. doi: 10.3390/biomedicines12030551 (PMC10968290; doi:10.3390/biomedicines12030551)
Supplement: Supplementary file 1 [file biomedicines-12-00551-s001.zip › biomedicines-2885900-supplementary.pdf]

Hyperchloremia and Hypernatremia Decreased Microglial and Neuronal Survival during Oxygen–Glucose Deprivation/Reperfusion

Reetika Mahajan [1], Faheem Shehjar [1], Adnan I. Qureshi [2] and Zahoor A. Shah [1,*]

[1] Department of Medicinal and Biological Chemistry, College of Pharmacy and Pharmaceutical Sciences, University of Toledo, Toledo, OH 43614, USA

[2] Zeenat Qureshi Stroke Institutes and Department of Neurology, University of Missouri, Columbia, MO 65211, USA

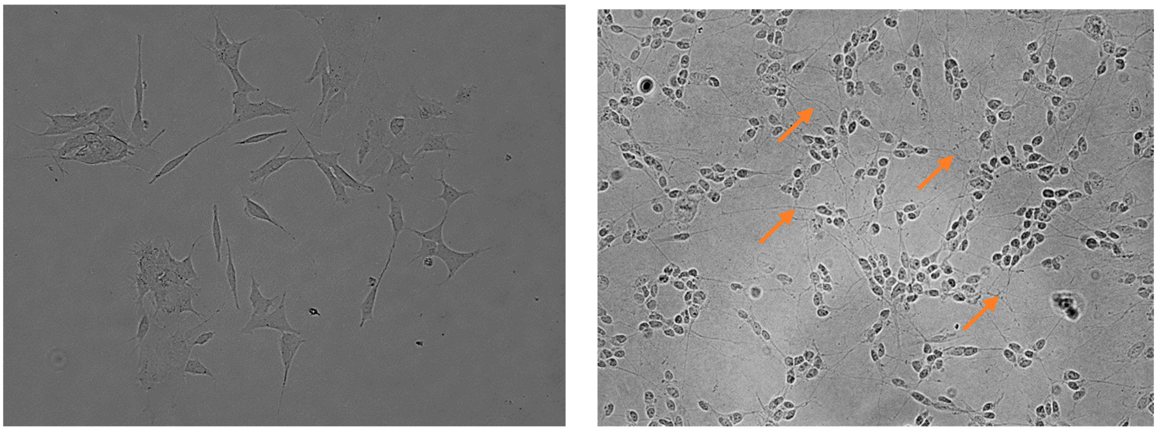

a. Undifferentiated SH-SY5Y cells

b. Differentiated SH-SY5Y cells (5 days after 10µM Retinoic acid treatment). Orange arrows represent the neurites.

Supplementary Figure S1: Showing the differentiation of SH-SY5Y neuroblastoma into neuron like cells. a. Undifferentiated SH-SY5Y cells b. fully differentiated SH-SY5Y cells (neuron like cells).
